# Supplementary figures and images for: Targeted next-generation sequencing: a promising approach for Mycobacterium tuberculosis detection and drug resistance when applied in paucibacillary clinical samples
Source: Microbiol Spectr. 2025 Jun 10;13(7):e03127-24. doi: 10.1128/spectrum.03127-24 (PMC12211088; doi:10.1128/spectrum.03127-24)

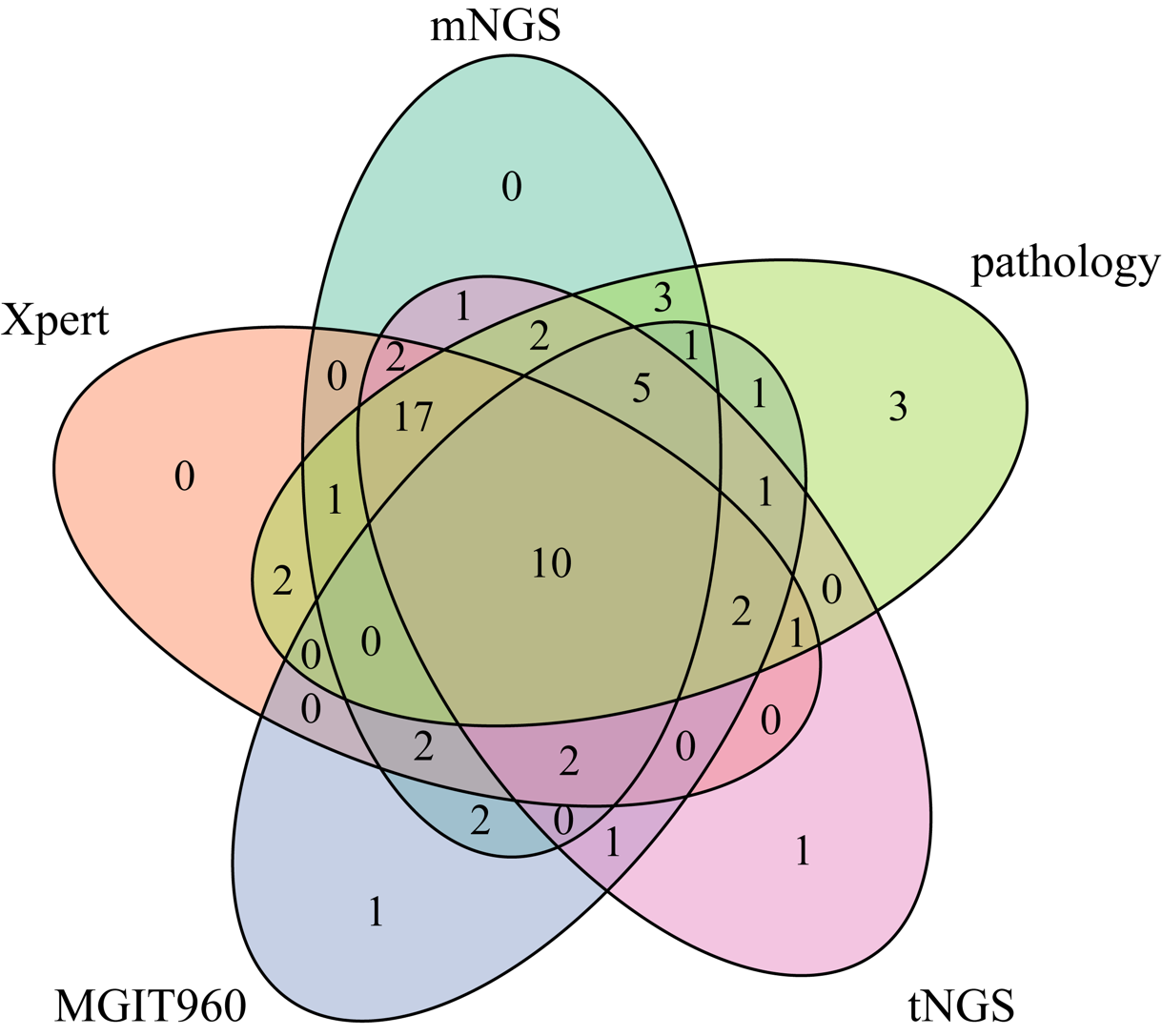

Supplement: Fig. S1 — Venn diagram of positive tests for 74 tissue samples. [file spectrum.03127-24-s0001.tif]
