# Supplementary material for: Targeted next-generation sequencing: a promising approach for Mycobacterium tuberculosis detection and drug resistance when applied in paucibacillary clinical samples
Source: Microbiol Spectr. 2025 Jun 10;13(7):e03127-24. doi: 10.1128/spectrum.03127-24 (PMC12211088; doi:10.1128/spectrum.03127-24)
Supplement: Supplemental material — Fig. S1 legend. [file spectrum.03127-24-s0002.docx]

Figure S: Venn diagramof positive tests for74 tissue samples.
