# Supplementary material for: Targeted next-generation sequencing: a promising approach for Mycobacterium tuberculosis detection and drug resistance when applied in paucibacillary clinical samples
Source: Microbiol Spectr. 2025 Jun 10;13(7):e03127-24. doi: 10.1128/spectrum.03127-24 (PMC12211088; doi:10.1128/spectrum.03127-24)
Supplement: Supplemental tables — Tables S1 to S4. [file spectrum.03127-24-s0003.docx]

**Table S1. The diagnostic performance of four methods for tuberculosis in diverse sample types in comparison to CRS standard**

| **CRS as standard** | **Method** | **Sensitivity (95% CI)** | **Specificity (95% CI)** | **Accuracy (95% CI)** | **AUC (95% CI）** | ***P* value** |
| --- | --- | --- | --- | --- | --- | --- |
| All (n=178) | culture | 0.458 (0.377-0.540) | 1.0000 | 0.547 (0.544-0.549) | 0.729 (0.688-0.770) | 0.000 |
|  | Xpert | 0.614 (0.534-0.695) | 1.0000 | 0.679 (0.676-0.681) | 0.807 (0.767-0.847) | 0.000 |
|  | mNGS | 0.772 (0.704-0.839) | 1.0000 | 0.808 (0.806-0.810) | 0.886 (0.852-0.920) | 0.000 |
|  | tNGS | 0.760 (0.692-0.828) | 1.0000 | 0.798 (0.796-0.800) | 0.880 (0.846-0.914) | 0.000 |
| Respiratory (n=104) | culture | 0.470 (0.363-0.577) | 1.0000 | 0.569 (0.564-0.573) | 0.735 (0.681-0.789) | 0.000 |
|  | Xpert | 0.646 (0.540-0.751) | 1.0000 | 0.714 (0.710-0.718) | 0.823 (0.770-0.876) | 0.000 |
|  | mNGS | 0.810 (0.726-0.893) | 1.0000 | 0.845 (0.842-0.847) | 0.905 (0.863-0.947) | 0.000 |
|  | tNGS | 0.765 (0.675-0.855) | 1.0000 | 0.808 (0.805-0.811) | 0.882 (0.837-0.927) | 0.000 |
| Non-respiratory (n=74) | culture | 0.443 (0.318-0.567) | 1.0000 | 0.514 (0.507-0.521) | 0.721 (0.659-0.784) | 0.006 |
|  | Xpert | 0.574 (0.450-0.698) | 1.0000 | 0.629 (0.622-0.635) | 0.787 (0.725-0.849) | 0.001 |
|  | mNGS | 0.723 (0.614-0.832) | 1.0000 | 0.757 (0.752-0.762) | 0.862 (0.807-0.916) | 0.000 |
|  | tNGS | 0.754 (0.649-0.859) | 1.0000 | 0.784 (0.779-0.788) | 0.877 (0.825-0.929) | 0.000 |
| Tissue (n=82) | culture | 0.470 (0.349-0.590) | 1.0000 | 0.545 (0.539-0.552) | 0.735 (0.675-0.795) | 0.002 |
|  | Xpert | 0.636 (0.520-0.752) | 1.0000 | 0.688 (0.683-0.694) | 0.818 (0.760-0.876) | 0.000 |
|  | mNGS | 0.775 (0.677-0.872) | 1.0000 | 0.805 (0.801-0.809) | 0.887 (0.839-0.936) | 0.000 |
|  | tNGS | 0.718 (0.614-0.823) | 1.0000 | 0.756 (0.752-0.761) | 0.859 (0.807-0.911) | 0.000 |
| Non tissue (96) | culture | 0.449 (0.338-0.559) | 1.0000 | 0.547 (0.542-0.552) | 0.724 (0.669-0.780) | 0.000 |
|  | Xpert | 0.595 (0.483-0.706) | 1.0000 | 0.670 (0.666-0.675) | 0.797 (0.741-0.853) | 0.000 |
|  | mNGS | 0.769 (0.676-0.863) | 1.0000 | 0.811 (0.807-0.814) | 0.885 (0.838-0.931) | 0.000 |
|  | tNGS | 0.797 (0.709-0.886) | 1.0000 | 0.833 (0.830-0.836) | 0.899 (0.854-0.943) | 0.000 |

Note: *P*<0.05 in AUC compared to CRS;

CI: confidence interval; ACC: accuracy; AUC: area under the curve; BALF: bronchoalveolar lavage fluid

**Table S2. The diagnostic performance of four methods for tuberculosis in diverse sample types in comparison to MRS standard**

| **MRS as standard** | **Method** | **Sensitivity (95% CI)** | **Specificity (95% CI)** | **Accuracy (95% CI)** | **AUC (95% CI）** | ***P* value** |
| --- | --- | --- | --- | --- | --- | --- |
| All (n=178) | culture | 0.606 (0.514-0.697) | 1.000 | 0.750 (0.748-0.752) | 0.803 (0.757-0.849) | 0.000 |
|  | Xpert | 0.811 (0.737-0.886) | 1.000 | 0.881 (0.880-0.882) | 0.906 (0.868-0.943) | 0.000 |
|  | mNGS | 0.856 (0.791-0.921) | 0.697 (0.586-0.808) | 0.797 (0.795-0.798) | 0.776 (0.688-0.865) | 0.000 |
|  | tNGS | 0.884 (0.825-0.943) | 0.773 (0.672-0.874) | 0.843 (0.841-0.844) | 0.828 (0.748-0.909) | 0.000 |
| Respiratory (n=104) | culture | 0.619 (0.499-0.739) | 1.000 | 0.765 (0.761-0.768) | 0.810 (0.750-0.869) | 0.000 |
|  | Xpert | 0.836 (0.743-0.929) | 1.000 | 0.898 (0.896-0.900) | 0.918 (0.872-0.964) | 0.000 |
|  | mNGS | 0.889 (0.811-0.966) | 0.7 (0.558-0.842) | 0.816 (0.813-0.818) | 0.794 (0.685-0.904) | 0.000 |
|  | tNGS | 0.875 (0.794-0.956) | 0.775 (0.646-0.904) | 0.837 (0.834-0.839) | 0.825 (0.720-0.930) | 0.000 |
| Non-respiratory (n=74) | culture | 0.587 (0.445-0.729) | 1.000 | 0.729 (0.723-0.734) | 0.793 (0.722-0.865) | 0.000 |
|  | Xpert | 0.778 (0.656-0.899) | 1.000 | 0.857 (0.854-0.861) | 0.889 (0.828-0.950) | 0.000 |
|  | mNGS | 0.812 (0.702-0.923) | 0.692 (0.515-0.870) | 0.770 (0.766-0.775) | 0.752 (0.608-0.896) | 0.000 |
|  | tNGS | 0.896 (0.809-0.982) | 0.769 (0.607-0.931) | 0.851 (0.848-0.855) | 0.833 (0.708-0.957) | 0.000 |
| Tissue (n=82) | culture | 0.574 (0.442-0.706) | 1.000 | 0.701 (0.696-0.707) | 0.787 (0.721-0.853) | 0.000 |
|  | Xpert | 0.778 (0.667-0.889) | 1.000 | 0.844 (0.841-0.848) | 0.889 (0.833-0.944) | 0.000 |
|  | mNGS | 0.825 (0.726-0.923) | 0.680 (0.497-0.863) | 0.780 (0.776-0.785) | 0.752 (0.611-0.893) | 0.000 |
|  | tNGS | 0.825 (0.726-0.923) | 0.840 (0.696-0.984) | 0.829 (0.826-0.833) | 0.832 (0.711-0.954) | 0.000 |
| Non tissue (96) | culture | 0.636 (0.509-0.763) | 1.000 | 0.789 (0.786-0.793) | 0.818 (0.755-0.882) | 0.000 |
|  | Xpert | 0.846 (0.748-0.944) | 1.000 | 0.912 (0.910-0.914) | 0.923 (0.874-0.972) | 0.000 |
|  | mNGS | 0.889 (0.805-0.973) | 0.707 (0.568-0.847) | 0.811 (0.807-0.814) | 0.798 (0.687-0.910) | 0.000 |
|  | tNGS | 0.945 (0.885-1.000) | 0.732 (0.596-0.867) | 0.854 (0.852-0.857) | 0.839 (0.741-0.936) | 0.000 |

Note: *P*<0.05 in AUC compared to MRS;

CI: confidence interval; ACC: accuracy; AUC: area under the curve; BALF: bronchoalveolar lavage fluid

**Table S3. The diagnostic performance of combination of Xpert and tNGS for tuberculosis in diverse sample types in comparison to CRS standard**

| **CRS as standard** | **Sensitivity (95% CI)** | **Specificity (95% CI)** | **Accuracy (95% CI)** | **AUC (95% CI）** | ***P* value** |
| --- | --- | --- | --- | --- | --- |
| All (n=178) | 0.800 (0.734-0.866) | 1.000 | 0.833 (0.832-0.835) | 0.900 (0.867-0.933) | 0.000 |
| Respiratory (n=104) | 0.810 (0.724-0.897) | 1.000 | 0.847 (0.844-0.850) | 0.905 (0.862-0.948) | 0.000 |
| Non-respiratory (n=74) | 0.787 (0.684-0.89) | 1.000 | 0.814 (0.81-0.819) | 0.893 (0.842-0.945) | 0.000 |
| Tissue (n=82) | 0.803 (0.707-0.899) | 1.000 | 0.831 (0.828-0.835) | 0.902 (0.854-0.949) | 0.000 |
| Non-tissue (96) | 0.797 (0.706-0.889) | 1.000 | 0.835 (0.832-0.838) | 0.899 (0.853-0.944) | 0.000 |
| Sputum (n=33) | 0.955 (0.868-1.000) | 1.000 | 0.966 (0.963-0.968) | 0.977 (0.934-1.000) | 0.000 |
| BALF (n=17) | 0.750 (0.505-0.995) | 1.000 | 0.812 (0.794-0.831) | 0.875 (0.752-0.998) | 0.007 |
| Pulmonary tissue (n=28) | 0.783 (0.614-0.951) | 1.000 | 0.815 (0.804-0.826) | 0.891 (0.807-0.976) | 0.002 |
| Extrapulmonary tissue (n=55) | 0.795 (0.676-0.915) | 1.000 | 0.824 (0.818-0.829) | 0.898 (0.838-0.957) | 0.000 |
| Serous effusion (n=34) | 0.714 (0.547-0.882) | 1.000 | 0.765 (0.754-0.775) | 0.857 (0.773-0.941) | 0.001 |

Note: *P*<0.05 in AUC compared to CRS; Eleven pus was not listed separately in the list of third classification because of the small sample size.

CI: confidence interval; ACC: accuracy; AUC: area under the curve; BALF: bronchoalveolar lavage fluid

**Table S4. The diagnostic performance of combination of Xpert and tNGS for tuberculosis in diverse sample types in comparison to MRS standard**

| **MRS as standard** | **Sensitivity (95% CI)** | **Specificity (95% CI)** | **Accuracy (95% CI)** | **AUC (95% CI）** | ***P* value** |
| --- | --- | --- | --- | --- | --- |
| All (n=178) | 0.934 (0.887-0.981) | 0.881 (0.88-0.882) | 0.790 (0.689-0.892) | 0.862 (0.788-0.936) | 0.000 |
| Respiratory (n=104) | 0.934 (0.872-0.997) | 0.888 (0.886-0.89) | 0.811 (0.685-0.937) | 0.873 (0.778-0.967) | 0.000 |
| Non-respiratory (n=74) | 0.933 (0.860-1.000) | 0.871 (0.868-0.875) | 0.760 (0.593-0.927) | 0.847 (0.727-0.967) | 0.000 |
| Tissue (n=82) | 0.907 (0.830-0.985) | 0.883 (0.88-0.886) | 0.826 (0.671-0.981) | 0.867 (0.751-0.983) | 0.000 |
| Non-tissue (96) | 0.962 (0.909-1.000) | 0.879 (0.877-0.881) | 0.769 (0.637-0.901) | 0.865 (0.773-0.958) | 0.000 |
| Sputum (n=33) | 1.000 | 1.000 | 1.000 | 1.000 | 0.000 |
| BALF (n=17) | 0.889 (0.684-1.000) | 0.875 (0.862-0.888) | 0.857 (0.598-1.000) | 0.873 (0.641-1.000) | 0.002 |
| Pulmonary tissue (n=28) | 0.900 (0.769-1.000) | 0.926 (0.921-0.931) | 1.000 | 0.950 (0.884-1.000) | 0.000 |
| Extrapulmonary tissue (n=55) | 0.912 (0.816-1.000) | 0.863 (0.858-0.867) | 0.765 (0.563-0.966) | 0.838 (0.690-0.987) | 0.000 |
| Serous effusion (n=34) | 0.923 (0.778-1.000) | 0.735 (0.724-0.747) | 0.619 (0.411-0.827) | 0.771 (0.595-0.947) | 0.001 |

Note: *P*<0.05 in AUC compared to MRS; Eleven pus was not listed separately in the list of third classification because of the small sample size.

CI: confidence interval; ACC: accuracy; AUC: area under the curve; BALF: bronchoalveolar lavage fluid
